# Production and characterization of murine models of classic and intermediate maple syrup urine disease

**DOI:** 10.1186/1471-2350-7-33

**Published:** 2006-03-31

**Authors:** Gregg E Homanics, Kristen Skvorak, Carolyn Ferguson, Simon Watkins, Harbhajan S Paul

**Affiliations:** 1 Departments of Anesthesiology and Pharmacology, University of Pittsburgh, Pittsburgh, PA 15261, USA; 2 Graduate Program in Molecular Genetics and Biochemistry, University of Pittsburgh, Pittsburgh, PA 15261, USA; 3 Department of Cell Biology and Physiology, University of Pittsburgh, Pittsburgh, PA 15261, USA; 4 Biomed Research & Technologies, Inc., Wexford, PA 15090, USA

## Abstract

**Background:**

Maple Syrup Urine Disease (MSUD) is an inborn error of metabolism caused by a deficiency of branched-chain keto acid dehydrogenase. MSUD has several clinical phenotypes depending on the degree of enzyme deficiency. Current treatments are not satisfactory and require new approaches to combat this disease. A major hurdle in developing new treatments has been the lack of a suitable animal model.

**Methods:**

To create a murine model of classic MSUD, we used gene targeting and embryonic stem cell technologies to create a mouse line that lacked a functional E2 subunit gene of branched-chain keto acid dehydrogenase. To create a murine model of intermediate MSUD, we used transgenic technology to express a human E2 cDNA on the knockout background. Mice of both models were characterized at the molecular, biochemical, and whole animal levels.

**Results:**

By disrupting the E2 subunit gene of branched-chain keto acid dehydrogenase, we created a gene knockout mouse model of classic MSUD. The homozygous knockout mice lacked branched-chain keto acid dehydrogenase activity, E2 immunoreactivity, and had a 3-fold increase in circulating branched-chain amino acids. These metabolic derangements resulted in neonatal lethality. Transgenic expression of a human E2 cDNA in the liver of the E2 knockout animals produced a model of intermediate MSUD. Branched-chain keto acid dehydrogenase activity was 5–6% of normal and was sufficient to allow survival, but was insufficient to normalize circulating branched-chain amino acids levels, which were intermediate between wildtype and the classic MSUD mouse model.

**Conclusion:**

These mice represent important animal models that closely approximate the phenotype of humans with the classic and intermediate forms of MSUD. These animals provide useful models to further characterize the pathogenesis of MSUD, as well as models to test novel therapeutic strategies, such as gene and cellular therapies, to treat this devastating metabolic disease.

## Background

Maple Syrup Urine Disease (MSUD) is a genetic disorder caused by a deficiency of branched-chain keto acid dehydrogenase (BCKDH), a mitochondrial multienzyme complex responsible for the oxidative decarboxylation of branched-chain keto acids derived from branched-chain amino acids (BCAA), leucine, isoleucine and valine (for review, see: [1]).

Patients with MSUD, depending on the mutation, show variable degrees of enzyme deficiency leading to several different clinical phenotypes [1]. Approximately 75% of MSUD patients have the classic form of the disease with BCKDH activity in the range of 0–2% of normal [1]. These patients show markedly elevated levels of BCAA in blood and other body fluids [1]. Besides the classic form, there are other variants of the disease. Patients with the intermediate form of the disease show BCKDH activity in the range of 3–30% of normal. In such patients the onset of the disease is delayed, but there are persistently elevated levels of BCAA [1]. Patients with the intermittent form of MSUD show BCKDH activity in the range of 5–20% and during the asymptomatic phase the blood BCAA levels are normal [1]. The overall incidence of MSUD in the general population is 1:185,000 [1], and in certain population groups, such as Mennonites of Pennsylvania, the incidence is estimated to be as high as 1:176 [2].

The BCKDH complex, the deficient enzyme in MSUD, consists of three catalytic proteins, a decarboxylase (E1), a dihydrolipoyl transacylase (E2), and a dihydrolipoyl dehydrogenase (E3). The E1 component is a heterotetramer composed of two α and two β subunits [3]. The E1 and E2 components are specific to BCKDH, whereas E3 is also used by pyruvate and α-ketoglutarate dehydrogenase complexes [3] and the glycine cleavage system [4]. BCKDH is also associated with two regulatory proteins, a specific kinase and a phosphatase which regulate the activity of this enzyme through a phosphorylation (inactivation) and dephosphorylation (activation) cycle of the E1 α subunit [5,6]. Mutations in the genes of the E1 and E2 subunits of BCKDH have been described, however, the majority of MSUD mutations identified thus far are in the E2 subunit [1,7]. To date, cases of MSUD have not been associated with defects in the regulatory kinase and phosphatase [1].

Current management of MSUD patients relies on a strict lifelong dietary restriction of protein or BCAA [1,8]. Such a dietary management of the disease, however, is not entirely satisfactory especially in times of metabolic decompensation due to infection, injuries and other stressors. In spite of dietary intervention, there is significant mortality associated with MSUD and there is a high incidence of mental retardation in survivors [9].

Because of the central role of the liver in amino acid metabolism and moderate/high levels of BCKDH activity in human liver [10-12], a few cases of MSUD have recently been treated by liver transplantation [13-16]. While the short-term outcome of liver transplantation is encouraging, long-term effects of this approach are not known. However, these patients are now required to take immunosuppressant drugs for the rest of their lives, often with undesirable side effects. Moreover, the cost associated with liver transplantation and the availability of donor livers are additional limiting factors for the practicality of treatment of this disease.

Because of the current unsatisfactory options for the treatment of MSUD, there is a need for improved therapies to combat this disease. An obstacle to developing novel treatments for MSUD has been the lack of a suitable animal model to perform necessary preclinical studies. Although a Hereford calf model with MSUD has been described [17-19], this model is neither readily available nor practical to perform preclinical studies. Furthermore, comparison of this animal model with human MSUD has shown some differences in the pathology of the disease [1], making this animal a less desirable model for the human disease.

Recently, a N-ethyl-N-nitrosourea (ENU)-induced mutant mouse that phenotypically resembles human MSUD has been described [20]. However, the mutation in this model disrupts a splice site in the mitochondrial branched-chain aminotransferase (BCAT) gene, not in BCKDH, the deficient enzyme in MSUD. Because the mutation is not in BCKDH, the validity of this mutant mouse line for modeling human MSUD is questionable.

The objective of the present study was to create genetically engineered murine models of MSUD that mimic the pathology of the classic and intermediate variant forms of the human disease. The classic model was created by targeted inactivation of the E2 subunit of BCKDH by homologous recombination in embryonic stem (ES) cells. The model of intermediate MSUD was created by partial transgenic rescue of the E2 gene knockout. This report describes the generation and characterization of these murine models of MSUD. These models will allow for the development of novel treatment approaches, such as gene or stem cell therapies, to ultimately cure MSUD.

## Methods

All studies involving animals were reviewed and approved by the University of Pittsburgh's Institutional Animal Care and Use Committee.

### Gene knockout production

**Figure 1:**
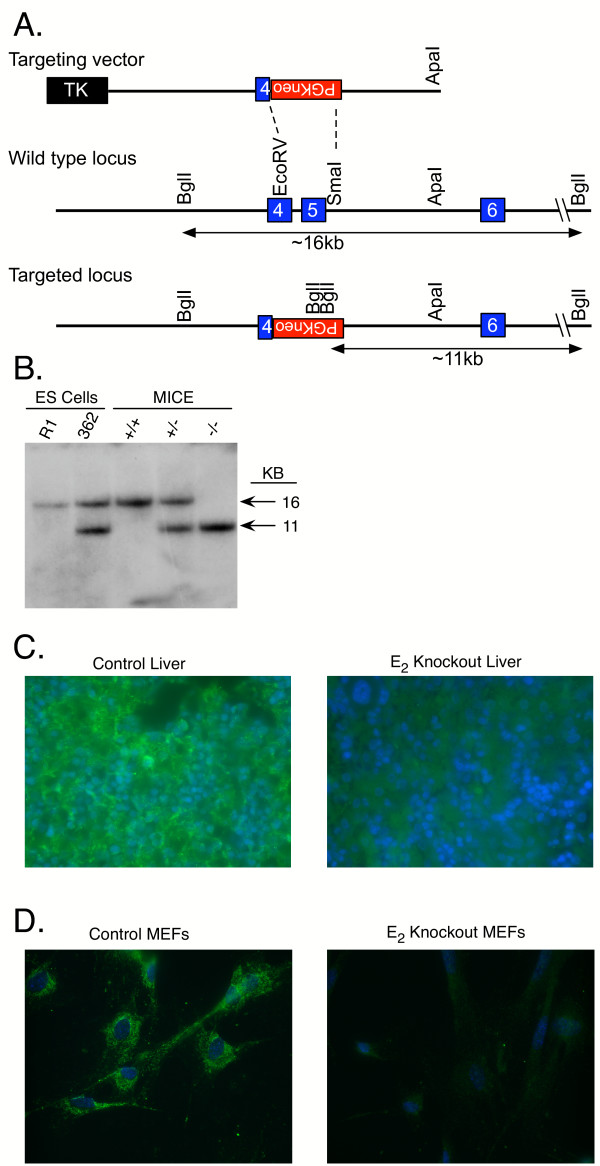
E2 gene knockout mouse production. *A*, Gene targeting strategy used for targeting the E2 locus in mouse ES cells. The targeting construct was designed to delete 1.67 kb of sequence between an EcoRV site in Exon 4 and a Smal site in intron 5. The wild type E2 gene contains an ~16 kb BglI restriction fragment that hybridizes to the Exon 6 specific probe. A correctly targeted E2 locus harbors an ~11 kb BglI restriction fragment that hybridizes to the same probe. Note that the probe will not detect random integration of the targeting vector because it is external to the targeting vector. *B*, Southern blot analysis of Bgll digested genomic DNA derived from the parental wild type ES cell line (R1), a heterozygous targeted ES cell line (362), and from wild type (+/+), heterozygous (+/-) and homozygous knockout (-/-) mice. The blot was hybridized with an Exon 6 specific probe. *C*, Immunohistochemical analysis of fresh frozen liver sections from control (+/+) and E2 knockout (-/-) postnatal day 1 mouse pups. Sections were stained for E2 using an E2 specific antibody (green) and a nuclear stain (blue). Note the complete absence of E2 immunoreactivity in the section from the knockout mouse. *D*, Similar results were observed upon immunohistochemical analysis of primary mouse embryonic fibroblasts (MEFs). Note that the readily detectable signal for E2 in the control cells was present in a pattern characteristic of mitochondria, the subcellular location of BCKDH.

A genomic DNA subclone of Strain 129/SvJ DNA that contained a portion of Exons 4 and 5 of the E2 gene and flanking DNA was obtained from a P1 phage library from Genome Systems, Inc., (St. Louis, MO; '3-Hit Mouse ES Library'). A positive/negative replacement type gene targeting vector [21] was created by replacing a 1.67 kb EcoRV-Smal fragment that included a portion of Exon 4 and all of Exon 5 with the PGKneo marker gene (See Fig. 1A) from the pPNT vector [22]. The targeting construct was linearized with NotI and electroporated into R1 ES cells [23] under conditions described previously [24]. ES cells were selected with G418 (300 μg/ml; Life Technologies Inc., Gaithersburg, MD) and gancyclovir (2 μM; gift of Syntex, Palo Alto, CA). Doubly resistant clones were screened for gene targeting by Southern blot analysis of BglI digested genomic DNA. Blots were hybridized with an Exon 6 specific probe that was external to the gene targeting construct. Correctly targeted ES cells were injected into C57BL/6J blastocysts to produce chimeric mice using standard procedures. Heterozygous offspring from germline competent chimeras were intercrossed to produce wild type (+/+), heterozygous (+/-) and homozygous knockout (-/-) mice. At all generations, +/- breeding pairs were used. Results presented here are from mice derived from the F2+ generations. All animals were of a mixed C57BL/6J × Strain 129Sv/SvJ genetic background.

### Production of transgenic mice

Standard molecular techniques were used to assemble the transgenic construct, pTRE-E2. This transgene contains the tetracycline responsive hCMV*^-1 ^promoter [consisting of the tetracycline responsive element (TRE) and a minimal hCMV promoter] [25] from pTRE2 (Clontech Inc., Mt. View, CA), a chimeric intron from pCI (Promega) to increase message stability and expression [26,27], a Kozak consensus sequence at the initiation codon to optimize translation [28], the human E_2 _cDNA which has been modified to contain a 4× alanine linker followed by a *c-myc *epitope tag at the carboxy terminus to facilitate detection, and an SV40 late polyadenylation sequence from pCI for enhanced mRNA stability and translation.

The 2.48 kb TRE-E2 transgene was purified from vector sequences following digestion with XhoI and BamHI and injected into C57BL/6J or C57BL/6J × Strain 129SvEv mouse embryos at the transgenic core facilities of the University of Pittsburgh and the University of Cincinnati, respectively. Genomic DNA from the tail of mice was screened by Southern blot analysis following digestion with EcoRI and hybridization to an ~400 bp probe derived from the SV40 portion of the TRE-E2 transgene.

### Production of intermediate MSUD murine model

The various TRE-E2 transgenic lines produced were mated independently to mice that were heterozygous for both the E2 knockout and the LAP-tTA transgene [Tg(tTALap)Bjd/J; Stock 3272; The Jackson Laboratory, Bar Harbor, ME; NMRI × FVB × C57BL/6J background]. Interbreeding of animals that were heterozygous for both transgenes and the knockout resulted in the production of mice with a variety of genotypes including some animals that were homozygous for the knockout and were positive for both transgenes (we refer to this genotype as the "rescue" genotype). If our strategy for rescuing the neonatal lethal phenotype of the knockout were successful, then those homozygous knockout animals that had both transgenes would survive beyond the neonatal period.

### Genotyping

All mice were genotyped by Southern blot analysis. Genomic DNAs prepared from tail snips were digested with an appropriate restriction enzyme, size fractionated by agarose gel electrophoresis, blotted to nylon, and probed using standard procedures.

### Immunohistochemistry

Primary mouse embryonic fibroblasts were prepared from embryonic day ~16.5–18.5 fetuses as described [29]. Fibroblasts were passed onto glass, fixed in 2% paraformaldehyde in PBS for 10 minutes, permeabilized in 2% paraformaldehyde containing 0.1% Triton X100 for 10 minutes and washed three times in PBS containing 0.5% BSA and 0.15% glycine, pH 7.4 (Buffer A). Following a 30 min incubation with purified goat IgG (50 (μg/ml) at 25°C and three additional washes with Buffer A, cells were incubated for 60 min with E2-specific antiserum [30] at 1 μg/ml followed by three washes in Buffer A and 60 minute incubation in fluorescently labeled second antibody (1–2 μg/ml). The cells were then washed six times (5 min/wash) in Buffer A and then mounted in gelvatol (Monsanto, St Louis). When livers from newborn pups were examined, fixation was by immersion in 2% paraformaldehyde followed by cryoprotection and shock freezing in liquid nitrogen cooled isopentane and sectioning (5 microns). Otherwise processing was as for the cells above (without the fixation and permabilization steps).

### Amino acid analysis

Blood was collected from the retroorbital sinus or tail vein of mice and spotted on a filter paper routinely used for blood amino acid analysis for prenatal screening. Concentrations of BCAA and other amino acids in blood were determined by tandem mass spectrometry (Pediatrix Screening, Bridgeville, PA).

### Assay of BCKDH activity

Livers were removed, frozen in liquid nitrogen, and stored at -80°C. At the time of enzyme assay, livers were thawed, and homogenized (1:9, w/v) in 0.25 M sucrose, 10 mM Tris-HC1, pH 7.4. Liver homogenates were centrifuged at 600 × g for 10 min at 4°C and the supernatant fraction was saved to determine the BCKDH activity. The use of tissue homogenates was necessitated by the limited availability of liver tissue, particularly from newborn pups. BCKDH activity was determined by measuring the release of ^14^CO_2 _from α-keto [1-^14^C] isocaproate as described previously [31]. The complete reaction mixture contained (final volume 1 ml) 30 mM potassium phosphate buffer, pH 7.4, 0.20 mM α-ketoisocaproate, 0.40 mM CoASH, 0.40 mM thiamin pyrophosphate, 2 mM NAD^+^, 2 mM dithiothreitol, 5 mM Mg^2+^, approximately 250,000 DPM of α-keto [1-^14^C] isocaproate, and 0.10 ml of liver homogenate (2–3 mg protein). Assays were carried out for 15 min at 37°C, ^14^CO_2 _was trapped in hydroxide of Hyamine, and radioactivity was determined by liquid scintillation spectrometry.

### Western blot analysis

Protein extracts were isolated from homogenized liver (freshly harvested and flash frozen) of wildtype, Line 525A, and Line A transgenic mice. Protein (25 μg per sample) was analyzed by electrophoresis on a 10% SDS-PAGE Ready Gel (Bio-Rad, Hercules, CA) and transferred to PVDF membrane (Sequi-Blot; Bio-Rad) via electroblotting. All blots were probed for E2 protein using polyclonal rabbit E2 antisera (1:5,000), which detects both mouse (~47 kD) and human (~54 kD) E2 subunits ([30]; gift from Dr. Susan Hutson, Wake Forest University). Blots were re-probed with a rabbit *anti-c-myc *tag antibody (1:10,000; abCam, Cambridge, MA; cat.# ab9106-100). Blots were also re-probed with an antibody for β-actin (43 kD; 1:10,000; abCam; cat.# ab8227-50) to allow for loading comparisons.  A goat anti-rabbit secondary antibody conjugated to horseradish peroxidase (1:10,000; Novus, Littleton, CO; cat.# NB730-H) was used for detection using the Western Lightning chemiluminescence reagent (Perkin Elmer, Boston, MA) and exposed to X-ray film.

### Statistical analyses

All BCAA and BCKDH enzyme activity data are presented as the mean +/- the standard error of the mean (S.E.M.). Differences between genotypes were compared by Student's t test[32].

## Results

### Production of E2 gene knockout mice

To create E2 knockout mice, we used gene targeting in mouse ES cells to disrupt the E2 gene. The overall strategy for disrupting the E2 gene is illustrated in Fig. 1A. The gene targeting construct was designed to replace a 1.67 kb EcoRV/Smal genomic DNA fragment encompassing part of Exon 4 and all of Exon 5 with the PGKneo selectable marker cassette. Of 522 ES cell clones screened for targeting by Southern blot analysis, 29 (5%) displayed the predicted restriction fragment length polymorphisms indicative of gene targeting at the E2 locus. As illustrated in Fig. 1A and 1B, an E2 Exon 6 specific probe, which is external to the targeting construct, hybridizes to only a ~16 kb BglI restriction fragment from the wild type allele in the parental R1 ES cell line. In correctly targeted ES cells, this probe also hybridizes to a ~11 kb BglI restriction fragment. Targeting was confirmed with several additional restriction enzymes and probes (data not shown).

Correctly targeted ES cells were microinjected into blastocysts to produce chimeric mice. Chimerics were bred to C57BL/6J mice. Following germline transmission of the targeted allele, heterozygous mice were interbred to produce wild type (+/+), heterozygous (+/-) and homozygous (-/-) animals. Mice were genotyped by Southern blot analysis. The Exon 6 specific probe hybridized to only a ~16 kb BglI restriction fragment in +/+ mice, a ~11 kb BglI fragment in -/- mice, and to both of these fragments in +/- mice (Fig. 1B).

Mice homozygous for the E2 mutation were born at the expected frequency. Genotype analysis of pups derived from +/- by +/- matings revealed that +/+, +/-, and -/- mice were present at nearly the expected 1:2:1 frequency. Of the initial 60 animals genotyped, 19 (32%) were +/+, 27 (45%) were +/-, and 14 (23%) were -/-. Thus, the E2 gene was dispensable for normal embryonic development. However, as expected, nearly all homozygous mice died in the perinatal period. Immediately following birth, homozygous pups were indistinguishable from their +/+ and +/- littermates; they were vigorous, active and able to suckle. By mid to late day on postnatal day one, most -/- pups became moribund and were readily identifiable as they were lethargic, pale, and exhibited gasping respiratory movements. With few exceptions, -/- pups died within 72 hours of birth. We have observed one rare -/- pup that survived to postnatal day 13. The reason for the prolonged survival of this pup is unknown.

### E2 deficient mice accurately model classic MSUD

**Figure 2:**
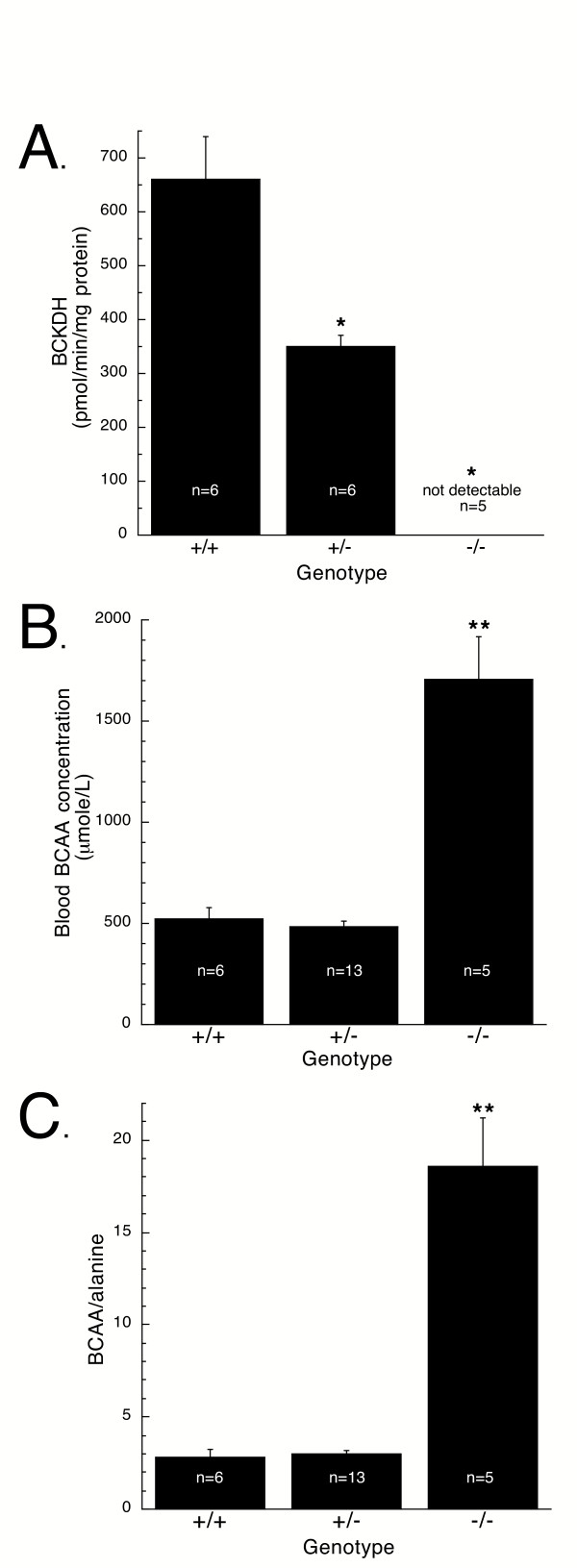
Biochemical characterization of the classic MSUD murine model. *A*, BCKDH enzyme activity in liver of newborn wild type control (+/+), heterozygous (+/-), and homozygous (-/-) knockout mice. Enzyme activity was significantly reduced in +/- liver compared to +/+, and was below the level of detection in -/- liver. B, Total BCAA concentrations in blood of mice. Total BCAA represent the sum of leucine, isoleucine, and valine. Total BCAA concentrations in blood from -/- mice were significantly elevated compared to +/+ and +/-. C, Ratio of total BCAA to alanine in blood of mice. This ratio was significantly elevated in -/- mice compared to +/+ and +/- mice. The numbers on the bars indicates the number of mice analyzed. *, Significantly different from +/+ (P < 0.001); **, significantly different from +/+ and +/- (P < 0.001).

To demonstrate that the gene targeting event created a true E2 null allele, we determined BCKDH activity in liver homogenates of postnatal day 1 mouse pups derived from +/- by +/- mating pairs. As shown in Figure 2A, the BCKDH activity in +/+ mice was readily detectable. In marked contrast, BCKDH activity was completely absent in -/- mouse livers. As expected, homogenates from +/- mice had approximately half the activity of their +/+ littermates (Figure 2A). The results from -/- mice are similar to that observed in humans with classic MSUD [1].

Immunohistochemistry with an E2 specific antibody was used to examine E2 protein in the mice. As shown in Figure 1C and 1D, immunoreactive E2 protein was abundant in liver and embryonic fibroblasts of+/+ mice. In marked contrast, immunoreactive E2 protein was absent in these same tissues of -/- mice.

Homozygous E2 knockout mice had a nearly 3-fold increase in blood (Figure 2B) and urine (data not shown) levels of BCAA (sum of leucine, isoleucine, and valine) as compared to their +/+ littermates. Because amino acids were analyzed by tandem mass spectrometry, the sum of BCAA shown in Figure 2B also may include alloisoleucine that may have been produced in -/- MSUD mice.

The metabolism of BCAA is linked with the synthesis of alanine, glutamate, and glutamine [33]. Because of impaired metabolism of BCAA in MSUD mice, and to further characterize the abnormal biochemistry in this model, we analyzed the blood levels of the alanine, glutamate, and glutamine. As shown in Table 1, the levels of all three amino acids in homozygous mice were markedly lower than the levels in +/+ or +/- mice. The levels of these amino acids in the +/- mice were comparable to those in +/+ mice (Table 1). Because of the abnormal decrease in the blood alanine level and marked rise in blood BCAA levels that are characteristic of MSUD, a recent report on MSUD patients has suggested that the ratio of BCAA/alanine provides a more sensitive measure of the abnormal biochemistry of MSUD than BCAA level alone [8]. Therefore, we also expressed the amino acid results as BCAA/alanine ratio. As shown in Figure 2C, this ratio in -/- pups was more than 6-fold higher than +/+ or +/- littermates. These blood amino acid results are consistent with the concentrations seen in patients with MSUD [1,8,15].

**Table 1:** Summary of additional blood amino acid levels in the classic MSUD model and control littermates as determined by tandem mass spectrometry. All samples were collected on the day of birth. All values are mean +/- SEM.

MSUD Genotype	Alanine	Glutamate	Glutamine
(μmole/L)			
+/+	196.1 ± 23.1 (6)	229.0 ± 12.0 (6)	76.8 ± 3.0 (6)
			
+/-	167.6 ± 9.9 (13)	251.0 ± 12.8 (13)	72.3 ± 1.6 (13)
			
-/-	94.6 ± 11.6* (5)	107.8 ± 4.8* (5)	50.4 ± 2.9* (5)

*: Significantly different from +/+ and +/- (P < 0.001).

In summary, E2 knockout mice lack BCKDH enzymatic activity, E2 immunoreactivity, and have markedly elevated levels of BCAA in the blood and urine. These metabolic derangements ultimately result in neonatal lethality. These phenotypes are remarkably similar to that observed in humans with the classic form of MSUD. Thus, E2 knockout mice closely model classic MSUD.

### Knockout mice expressing a human E2 transgene model a variant form of MSUD that mimics intermediate MSUD

**Figure 3:**
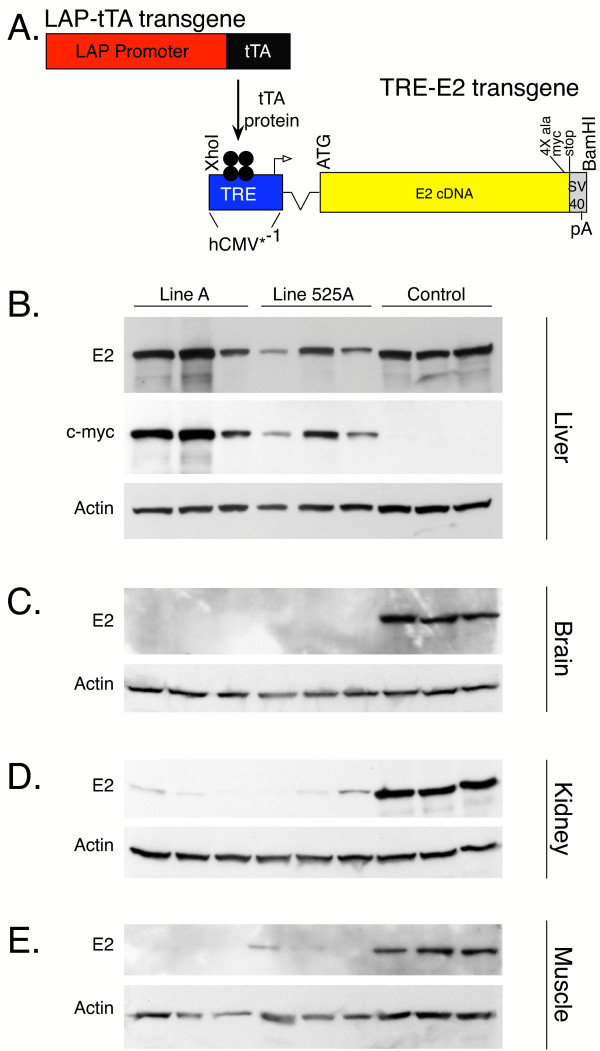
Transgenic mouse production and characterization. *A*, Transgenic strategy used to produce mice that express human E2. LAP-tTA transgenic mice have been previously described [34]. These mice express the tetracycline-controlled transactivator (tTA) from the liver specific LAP promoter. The TRE-E2 transgene contains the tetracycline response element (TRE) as part of the promoter, a synthetic intron (thin line), the human E_2 _cDNA, an alanine spacer, a *c-myc *epitope tag, and SV40 derived polyadenylation sequence. This construct was used to create several lines of transgenic mice. *B*, Western blot analysis of E2 protein in liver of control and intermediate MSUD mice. Note that the amount of human E2 protein (predicted MW ~54 Kd) in mice from Lines A and 525 A was variable but in many of the animals the amount was similar to the amount of mouse E2 (MW=~47 Kd) in control animals. Re-probing with a *c-myc *tag antibody confirmed the presence of the transgene derived, *c-myc *tagged, human E2 in transgenic mice but not in controls. Western blot analysis of brain (*C*), kidney (*D*), and muscle (*E*) revealed negligible amounts of transgene derived E2 in those tissues. All blots were re-probed with an actin antibody to allow amount of protein loaded in each lane to be compared.

To create a murine model of the intermediate variant form of MSUD, we used a transgenic strategy to rescue the severe elevation of BCAA and neonatal lethality that occurs in the classic MSUD mouse model. Our strategy was composed of a two part transgenic system to express human E2 in liver on the E2 knockout background. This bi-transgenic system consisted of a LAP-tTA transgene and a TRE-E2 transgene (Fig. 3A). The LAP-tTA transgene [34] directs high levels of liver specific expression of the tetracycline-controlled transactivator (tTA), a transcription factor that stimulates expression of promoters that harbor a transactivator response element (TRE). The TRE-E2 transgene was designed to express a human E2 cDNA from a TRE containing minimal promoter upon stimulation by tTA.

LAP-tTA mice were previously produced and characterized by the Bujard laboratory [34]. Pronuclear microinjection was used to produce transgenic mice that harbored the TRE-E2 transgene. From injections at the University of Pittsburgh, 2 transgene positive founders were identified that led to the generation of 3 different transgenic lines (Lines A, B, & D). From the injections at the University of Cincinnati, we obtained 8 transgene positive founders. Subsequent breeding of these founders revealed that many of the animals had multiple transgene insertion sites that segregated. We established a total of 15 different transgenic lines from these founders. Limited resources allowed us to only focus on a total of 9 of these lines. These lines differed substantially in transgene copy number as compared by Southern blot analysis of tail DNA (data not shown).

**Figure 4:**
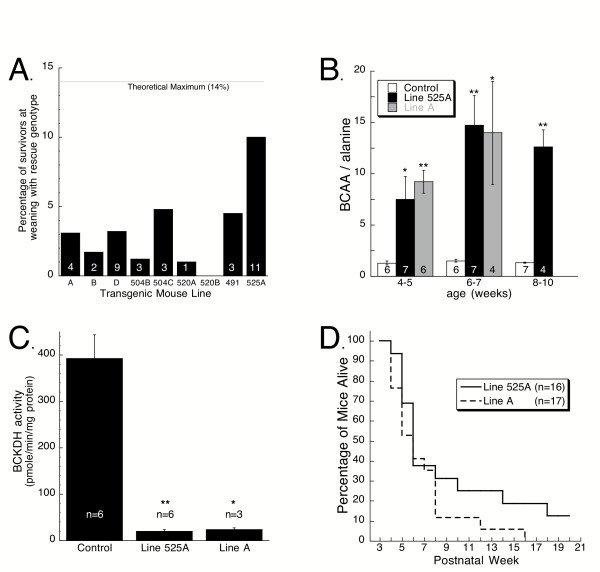
Characterization of the intermediate MSUD murine model. *A*, Survival analysis of transgenic rescue lines. Presented are percentages of mice alive at weaning from each transgenic line tested that had the rescue genotype (i.e., homozygous for knockout of endogenous E2 and positive for both the LAP-tTA and TRE-E2 transgenes). Also plotted is the theoretical maximum frequency at which this genotype is expected in this population of animals. The numbers on the bars indicates the numbers of observations for each line. *B*, Ratio of total BCAA to alanine in blood of mice from controls and Lines A and 525A. Total BCAA represent the sum of leucine, isoleucine, and valine. BCAA/alanine values were significantly greater for Lines A and 525A compared to controls at all ages tested. The numbers on the bars indicates the numbers of samples analyzed. *, P ≤ 0.01; **, P < 0.005. *C*, BCKDH enzyme activity in liver of control and Lines A and 525A mice. *, P ≤ 0.001; **, P ≤ 0.0001. *D*, Survival curves for mice from Lines A and 525A. Rescue mice alive at weaning were monitored until they were moribund and subsequently sacrificed or were found dead in their cages.

Transgenic TRE-E2 mice from each line (either founders or F1 offspring) were crossed to mice that were positive for the LAP-tTA transgene and were heterozygous for the E2 knockout. Ultimately, breeding pairs were used in which the mice were heterozygous for the LAP-tTA transgene, the TRE-E2 transgene, and the E_2 _knockout. To efficiently screen for the ability of the transgenes to rescue the knockout from neonatal lethality, we genotyped litters at weaning. This breeding strategy is expected to result in a theoretical maximum of animals with the rescue genotype (i.e., homozygous E2 knockout and positive for both transgenes) of 14%. Fig. 4A shows the percentage of mice alive at weaning with the rescue genotype from each transgenic line tested. Considerable variability was observed between lines. The line with the highest percentage of rescue mice at weaning was line 525 A. Approximately 10% of weaned, surviving pups from this line had the rescue genotype. Thus, the 525A transgene appears to be highly effective at rescuing the neonatal lethal phenotype of the E2 knockout. In contrast, line 520B completely failed to rescue the neonatal lethal phenotype. All other lines tested produced surviving rescue animals at a frequency between 1 and 5% of weaned pups.

Lines A and 525A were selected for detailed characterization. Fig. 4B shows the results of blood amino acid analysis presented as a ratio of total BCAA to alanine. The BCAA/alanine values for mice from both lines were significantly elevated compared to controls for all of the time points analyzed. Note that the BCAA/alanine values for the transgenic mice are intermediate between controls and knockouts (see Figure 2C). Blood levels of alanine, glutamate, and glutamine were reduced in Line A mice, as was glutamate in Line 525A mice, compared to controls (Table 2).

**Table 2:** Summary of additional blood amino acid levels in the intermediate MSUD model and littermate controls as determined by tandem mass spectrometry. Samples were collected from mice that were 4–6 weeks of age. All values are mean +/- SEM.

MSUD Genotype	Alanine	Glutamate	Glutamine
(μmole/L)			
control	225.2 ± 36.6 (11)	106.5 ± 10.5 (11)	67.8 ± 7.6 (11)
			
525A	175.0 ± 28.7 (10)	68.4 ± 7.7** (10)	58.0 ± 7.0 (10)
			
A	144.1 ± 16.9* (10)	65.9 ± 8.9** (10)	37.7 ± 6.5**,*** (10)

*: Significantly different from control (P < 0.05).

**: Significantly different from control (P < 0.01).

***: Significantly different from Line 525A (P < 0.05).

Because the LAP-tTA mice that were used to drive expression of transgenic human E2 have been demonstrated to produce liver specific expression [34], BCKDH enzyme activity and production of human E2 protein in liver of Lines A and 525A was examined. BCKDH enzymatic activity in liver from Lines A and 525A was ~6 and 5%, respectively, of the enzymatic activity present in control liver (Fig. 4C). As shown in Fig. 3B, the amount of human E2 (predicted MW~54 Kd) in these transgenic mice was quite variable between mice. In some of these transgenic mice, the level of human E2 was approximately equal to the amount of mouse E2 (~47 Kd) produced in liver of nontransgenic control mice. The observations that these near normal amounts of E2 protein result in only ~5–6% of normal BCKDH enzyme activity suggest that transgene derived E2 is functioning at a suboptimal level. It is probable that the *c-myc *tag at the carboxy terminus of the transgene derived human E2 interfered with enzymatic activity. This interpretation is consistent with previous studies which have revealed that the carboxy terminus of an analogous subunit of the pyruvate dehydrogenase complex is essential for subunit interactions [35] and insertion of a Hisx6 tag on the carboxy terminus of an analogous *E. coli *subunit interfered with normal subunit assembly [36]. It is also possible that human E2 was not fully functional when complexed with mouse E1 and E3 subunits. Human E2 shares ~88% identity to mouse E2 at the amino acid level. We also used western blot analysis to analyze expression of the E2 transgene in brain, kidney and muscle. As shown in Figure 3C, D and 3E respectively, expression of E2 is negligible in those tissues.

Long-term survival of the mice from these two transgenic lines that survived beyond weaning is plotted in Fig. 4D. Although survival data for control animals was not collected, it is readily apparent that survival of the rescue mice was compromised. By 16 weeks, all mice of Line A were moribund and humanely sacrificed, or were found dead in their cage. Survival of Line 525A appeared to be somewhat better. At 20 weeks of age, ~12% of mice (2 of 16) were still alive. These two rare survivors died at 40 and 60 weeks of age.

In summary, these surviving mice had BCAA/alanine ratios that were intermediate between controls and knockouts and they expressed ~5–6% of normal BCKDH enzyme activity in the liver. These phenotypic observations are remarkably similar to the clinical phenotype observed in MSUD patients with the intermediate form of the disease [1]. Thus, these rescue mice represent a very useful model of the intermediate MSUD phenotype.

## Discussion

A major hurdle in developing new treatments for MSUD has been the lack of a practical, accurate animal model of the disease. This hurdle has now been overcome. In this report, we describe the development and characterization of two genetically engineered mouse models that are phenotypically very similar to MSUD patients with the classic and intermediate forms of the disease.

Our model of classic MSUD was created by gene knockout of the E2 subunit of BCKDH. The phenotype of these knockout animals is strikingly similar to humans with classic MSUD. Knockout mice were born at the expected frequency and appeared normal at birth. Within a day of birth and following suckling, blood levels of BCAA were markedly elevated. Concomitantly, levels of the amino acids alanine, glutamate, and glutamine, whose synthesis is linked with normal metabolism of BCAA, were decreased (Table 1). The activity of BCKDH in livers of homozygous knockout mouse pups was undetectable, accounting for the accumulation of unmetabolized BCAA. Immunoreactive E2 protein was absent in liver and fibroblasts of homozygous pups. The phenotypic behavior of homozygous mouse pups resembles symptoms seen in newborn classic MSUD patients. These include signs of neurologic dysfunction such as seizures, stupor, lethargy, loss of motor activity, and respiratory difficulties [1]. These neurologic symptoms may result from reduced levels of glutamate, glutamine, alanine, and other similar neuroactive amino acids, which are considered the culprit for MSUD encephalopathies in patients [1,37]. Finally, nearly all of the homozygous pups died within 72 hours of birth. This neonatal lethality is likely due to the accumulation of BCAA to neurotoxic levels, ketoacidosis, brain edema, dehydration, and malnutrition as observed in the MSUD calf [17] and in classic MSUD patients [1]. Heterozygous knockouts were normal and had normal levels of BCAA despite having approximately half of BCKDH enzymatic activity. From the characterization studies completed thus far, the null mutation mouse accurately represents a model of classic MSUD and appears to be a faithful model of the human disease with respect to several biochemical phenotypes [1].

To create a model of intermediate MSUD, we used a transgenic strategy to express human E2 in the liver of E2 knockout mice. As mentioned above, E2 knockouts without transgene derived E2 die during the early neonatal period. We show that expression of a human E2 transgene in the liver of these knockout mice is able to rescue the neonatal lethality. Many of the rescue mice survived to adulthood. It is interesting to note that in these mouse lines only ~5–6% of normal BCKDH activity in the liver was sufficient to allow survival. We also demonstrated that BCAA levels in blood of these rescue mice were intermediate between controls and knockouts. The hallmarks of intermediate MSUD human patients are persistently increased levels of BCAA and BCKDH activity in the range of 3–30% of normal [1]. Because of the phenotypic similarities of the rescue mice to the human patients, these genetically engineered mice represent a useful small animal model of the intermediate form of MSUD.

The transgenic approach that was used to create the intermediate MSUD model was based on the tetracycline regulated gene switch system that has been used with great success in other studies, for example [34]. The strategy behind our approach was to create mice with an intermediate MSUD phenotype that could be converted to the classic phenotype at the investigator's discretion by turning off the rescuing transgene. However, we were unable to consistently turn off the human E2 expression cassette by the potent tetracycline analogue, doxycycline in either of the two transgenic lines tested (data not shown). The reason these two lines were unresponsive to doxycycline is unknown. It is conceivable that the integration site of the transgenes was not permissive for regulated expression. Screening of additional lines may be needed to find lines that allow for survival and can also be regulated.

The models described in this communication are important advances over the previously described models. An ENU mouse resembling human MSUD was previously created by disrupting the mitochondrial BCAT gene [20]. The BCKDH activity in the liver and muscle of this mouse was normal even though the blood BCAA levels were markedly elevated. While this is an interesting model, it is not a true model of MSUD because the mutation is not in BCKDH and the levels of BCKDH are normal. Furthermore, no case of MSUD has been described attributing this disease to BCAT deficiency. In addition to this mouse model, a cow model of MSUD has also been previously described [17-19]. However, due to practical constraints imposed by such a large animal model and due to the observation of differences between the MSUD cow model and MSUD humans [1], this model is also of limited utility. In contrast, the murine models described in this report are more valid and appropriate for modeling human MSUD.

The most significant opportunity presented by the MSUD mouse models is to test novel treatments such as gene [38-41] and cell based therapies (e.g., hepatocytes [42,43] or embryonic stem cells [44]). Relevant to gene therapy, recent problems with gene therapy in humans highlight the importance and critical need of animal models of genetic diseases for preclinical studies. The testing of gene and cell based therapies on appropriate animal model systems provides a preliminary method of establishing not only the efficacy, but also short- and long-term safety. Success with animal studies is expected to advance such therapeutic approaches and could pave the way for studies in humans. In addition to testing various treatments, these models should also be very useful for investigating the underlying pathophysiologic consequences of the disease. Such studies are very difficult/impossible to do in human patients for obvious ethical reasons, and often must rely on autopsy tissue. Additionally, the intermediate MSUD model may also be useful for studies to test the effect of thiamin. A thiamin-responsive form of MSUD has been described [1]. The phenotype of these patients is heterogeneous and treatment with a wide range of thiamin doses has produced limited success [1]. Recent cell culture studies with MSUD cells have suggested that the thiamin-responsive phenotype is dependent upon the presence of at least one E2 expressing allele [45,46]. In light of these newer findings, our intermediate MSUD mouse provides a model to test the effect of thiamin with respect to BCKDH activity and blood amino acid levels at the level of the whole animal. Because of recent interest in structural analysis of multienzyme complexes [35,36,45,46], the intermediate MSUD model may provide a valuable resource for studies of structural biology. Lastly, the information and knowledge gained from studies with the MSUD models described here will also be applicable and transferable to other mitochondrial disorders due to defects in multisubunit enzymes.

## Conclusion

In summary, this report describes the development and characterization of genetically engineered mouse models of classic as well as intermediate MSUD. These animals provide useful models to further characterize the pathogenesis of MSUD, as well as models to test novel therapeutic strategies, such as gene and cellular therapies, to treat this devastating metabolic disease.

## Abbreviations

The abbreviations used are: MSUD, Maple Syrup Urine Disease; BCKDH, branched chain ketoacid dehydrogenase; BCAA, branched chain amino acids; ENU, N-ethyl-N-nitrosourea; BCAT, branched chain aminotransferase; ES cell, embryonic stem cell; TRE, tetracycline responsive element; MEF, mouse embryonic fibroblast.

## Competing interests

The author(s) declare that they have no competing interests.

## Authors' contributions

GEH designed and produced the mouse models, coordinated experiments, interpreted data, and helped draft the manuscript. KS conducted much of the characterization of the intermediate MSUD model. CF assisted with production of the mouse models, data collection, analysis, and interpretation. SW directed the immunohistochemistry experiments. HSP conceived of the knockout model, interpreted data and drafted the manuscript. All authors contributed to composing and editing the manuscript and have read and approved the final version.

## Pre-publication history

The pre-publication history for this paper can be accessed here:


